# Alternatively activated macrophages; a double-edged sword in allergic asthma

**DOI:** 10.1186/s12967-020-02251-w

**Published:** 2020-02-05

**Authors:** Mohamed Hamed Abdelaziz, Sayed F. Abdelwahab, Jie Wan, Wei Cai, Wang Huixuan, Cheng Jianjun, Kesavan Dinesh Kumar, Aparna Vasudevan, Ahmed Sadek, Zhaoliang Su, Shengjun Wang, Huaxi Xu

**Affiliations:** 1 Department of Immunology, School of Medicine, Jiangsu University, Zhenjiang, 212013 Jiangsu China; 2 Department of Microbiology and Immunology, Faculty of Medicine, Minia University, Minia, 61511 Egypt; 3 Division of Pharmaceutical Microbiology, Department of Pharmaceutics and Pharmaceutical Technology, Taif University, College of Pharmacy, Taif, 21974 Kingdom of Saudi Arabia; 4 Department of Microbiology & Immunology, School of Medicine, Assiut University, Assiut, 71515 Egypt

**Keywords:** Allergy, Asthma, Human/mice, IL-4, Lung, Macrophages

## Abstract

**Background:**

Macrophages are heterogenous phagocytic cells with an important role in the innate immunity. They are, also, significant contributors in the adaptive immune system. Macrophages are the most abundant immune cells in the lung during allergic asthma, which is the most common chronic respiratory disease of both adults and children. Macrophages activated by Th1 cells are known as M1 macrophages while those activated by IL-4 and IL-13 are called alternatively activated macrophages (AAM) or M2 cells. AAM are subdivided into four distinct subtypes (M2a, M2b, M2c and M2d), depending on the nature of inducing agent and the expressed markers.

**Body:**

IL-4 is the major effector cytokine in both alternative activation of macrophages and pathogenesis of asthma. Thus, the role of M2a macrophages in asthma is a major concern. However, this is controversial. Therefore, further studies are required to improve our knowledge about the role of IL-4-induced macrophages in allergic asthma, through precisive elucidation of the roles of specific M2a proteins in the pathogenesis of asthma. In the current review, we try to illustrate the different functions of M2a macrophages (protective and pathogenic roles) in the pathogenesis of asthma, including explanation of how different M2a proteins and markers act during the pathogenesis of allergic asthma. These include surface markers, enzymes, secreted proteins, chemokines, cytokines, signal transduction proteins and transcription factors.

**Conclusions:**

AAM is considered a double-edged sword in allergic asthma. Finally, we recommend further studies that focus on increased selective expression or suppression of protective and pathogenic M2a markers.

## Background

### Macrophages: development, polarization and subsets

Macrophages are the major effector cells of the innate immune system that participate in the potent effector mechanism of the adaptive immune system. Macrophages were initially identified by Elie Metchnikoff who demonstrated the action of phagocytes in starfish larvae in 1883 [1]. Macrophages development occur during both early fetal development and adult life. They are derived from the yolk sac and fetal liver, generating heterogenous long-lived tissue resident macrophages that are widely distributed in different tissue and organs with diverse functions and subsets. These include Kupffer cells in the liver, microglial cells in the brain and alveolar macrophages in the lung. In adult life, macrophages are derived from bone marrow stem cells in response to monocyte colony stimulating factor to form monocytes (the precursor of macrophages), circulating in the blood. After initiation of inflammation, they migrate to inflammatory tissues and mature into macrophages and perform their function [2]. In this article, we are concerned about alveolar macrophage in human and mice.

Alveolar macrophages reside in the inner surface of the lung, accounting for 55% of lung immune cells, and can differentiate to major subsets in response to different stimuli. Unlike the second type of lung macrophage; interstitial macrophages, which reside in the interstitial areas of the lung, maintain homeostasis and induce tolerance for harmless antigens [3]. Generally, macrophages perform distinct functions depending on the type of exposed stimuli. IFN-γ, which was formerly called macrophage-activating factor, activates resting macrophages to kill ingested microbes by the action of nitric oxide (NO), reactive oxygen species and lysosomal enzymes. This activation is called classical macrophage activation as it was identified first and describes the classical pathway of activation by Th1 cells. They are known as M1 macrophages (named M1 to mirror Th1 nomenclature). IFN-γ is mainly secreted by Th1 cells; which is activated by IL-12 secreted by activated macrophages; reflects the synergism between Th1 and M1 macrophages. Also, this synergism occur through binding of macrophage molecules CD80/CD86 and CD40 with T cells’ CD28 and CD40L, respectively [4]. By contrast, IL-4 and IL-13 activate resting macrophages to an alternative form of macrophages, the so called alternative activated macrophages (AAM) or M2 macrophage (named M2 to mirror Th2 nomenclature), or anti-inflammatory macrophages. M2 polarization antagonizes M1 polarization; since IL-4 suppresses Th1 and M1 polarization. M2 cells antagonize the effects of M1 cells (mediated through IL-10), and promote tissue repair, remodeling and wound healing (through TGF-β and other factors) after inflammatory injury [4, 5]. This reflects the important role of M2 macrophages as a natural feedback regulator of the inflammatory process in the form of termination and repair.

Based on in vitro experiments, AAM are subdivided into four distinct subtypes [4, 6–8] (Table 1), namely M2a, M2b, M2c and M2d, depending on the nature of inducing agent and the expressed markers. Whether all subtypes are expressed in vivo, is still unclear [4, 7, 8]. In this review, we focus on human and mice M2a macrophages, which is induced by IL-4 and IL-13, expressing high CD206, Arg1, Ym1, FIZZ1 and TGF-β, promoting fibrosis and wound healing, so called wound healing macrophage [4, 6–8].

**Table 1:** M2 subsets of macrophages, inducing stimuli, significant markers and functions

M2 subtype	Inducing stimuli	Signature markers	Functions	References
M2a^a^	IL-4, IL-13 and M-CSF	CD206, Arg1, Ym1, FIZZ1 IL-10, TGF-β	Anti-inflammatory and Wound healing	[4, 6–8]
M2b	TLR ligands + IL-1R agonist	CD206, IL-1 β, IL-6, TNF-α, IL-12^Low^ IL-10	Immuno-regulation and promoting infections	
M2c	IL-10, Glucocorticoids, TGF-β	CD206, CD163, MerTK IL-10, TGF- β	Efferocytosis and tissue remodeling	
M2d	TLR + adenosine A2A R ligands, IL-6	VEGF, IL-10 TGF- β IL-12^Low^, TNF-α^Low^	Angiogenesis, Tumor growth	

^a^M2a macrophage is induced by IL-4 and IL-13, expressing high CD206, Arg1, Ym1, FIZZ1 and TGF-β, promoting fibrosis and wound healing, so called wound healing macrophage. M2b is stimulated by exposure to both immune complex and Toll like receptor (TLR) ligand or IL-1 receptor agonist. M2b is the only subtype that secrets proinflammatory cytokines; IL-1β, IL-6 and TNF-α, however it secrets low IL-12 (So not inducing Th1) and high anti-inflammatory IL-10, thus, performing some immunoregulatory functions. M2c is induced by IL-10, glucocorticoids and TGF-β, expressing high levels of innate receptors CD206, CD163 and the Mer receptor tyrosine kinase (MerTK) which enable it to perform efferocytosis function (phagocytic clearance of dead cells). M2d is induced by combined exposure to TLR with adenosine A2A receptor ligands, or by IL-6, expressing high vascular endothelial growth factor (VEGF) and IL-10, enabling it to induce angiogenesis and promote tumor growth

### Asthma: epidemiology and pathogenesis

Bronchial asthma is the most common chronic respiratory disease, with around 334 million people affected worldwide, with higher prevalence in developed countries [9]. The most common form of asthma is due to allergic stimuli, so called allergic or atopic asthma, while other minor forms are caused by non-allergic stimuli such as air pollution, cold, aspirin and exercise. However, the pathophysiologic processes of these types are almost the same [10]. Allergic asthma is caused by immediate hypersensitivity reaction (type I), which is initiated by antigen exposure, activating specific Th2 cells that produce IL-4, IL-5 and IL-13. Then, IL-4 stimulates B cells to secrete IgE which binds to Fcε receptors on mast cells and basophils, leading to their degranulation upon re-exposure to the same antigen. The degranulation process results in the release of preformed biogenic amines (histamine), granule enzymes and proteoglycans, stimulating bronchoconstriction and increasing vascular permeability followed by the release of newly synthesized lipid mediators (prostaglandin D2, leukotrienes and platelet-activating factor), that stimulate further bronchoconstriction and vascular permeability with chemotaxis of more inflammatory cells, in addition to cytokines’ released by mast cells (TNF, IL-1, IL-4, IL-5, IL-6, IL-13, CCL3, CCL4) that mediate late phase inflammatory reactions [9–11]. Concurrent to mast cells activation, eosinophils are activated by IL-5, which enhances eosinophilic maturation from bone marrow cells, recruitment to inflammatory sites and release of lipid mediators like mast cells and basophils. All previous factors lead to the characteristics of allergic asthma including airway obstruction, airway hyperreactivity (AHR) to specific stimuli, chronic infiltration and hypertrophy of bronchial smooth muscle cells [10, 11].

### Alternatively activated macrophages and allergic asthma

Since the macrophages are the predominant cells in the lung during allergic asthma [12, 13], and IL-4 is the key cytokine in both alternative activation of macrophages and pathogenesis of asthma [14], elucidating the role of M2a cells in asthma is a major concern. However, there is a controversy about this role. Some studies suggest that AAM increases the pathogenesis of asthma through promotion of allergic inflammation and AHR. Also, it induces airway remodeling through deposition of collagen supported by the correlation between increased M2 cells and severity of asthma, and by exacerbations induced with adoptive transfer of M2 cells suggesting that targeting them might be an efficient option for asthma treatment [15–17]. Others think that AAM doesn’t have a significant role in the developments of asthma, supported by a study showing that deletion of macrophage IL-4Rα doesn’t affect the pathology of allergic asthma, and increased M2 cell percentage in asthma is just an association of increased Th2 response [18]. Therefore, further studies are required for better understanding of the role of IL-4-induced AAM (M2a) in allergic asthma, and these studies should involve elucidation of the roles of specific M2 proteins in the pathogenesis of asthma, which was already demanded in 2011 [19]. Since then, no study emphasized the roles of M2a markers in allergic asthma.

In this review, we elucidate the diverse roles of M2a cells in the pathogenesis of asthma, through illustration of how its significant markers react during development of asthma. Some markers are not exclusive for macrophages. However, as mentioned earlier; macrophages are the predominant cells in asthma and thus their receptors and cytokines are suggested as higher contributors in asthma than other cells. Specifically, some macrophage major transcription factors are involved in the polarization of Th2 cells. Therefore, we will focus on the role of transcription factors in the induction of M2 polarization only, not generally in asthma, to avoid the over-estimation of transcription factors roles in the disease. According to markers’ nature, we divided M2a markers into six categories (Table 2), to simplify discussions about their roles in allergic asthma.

**Table 2:** M2a cell markers in human and mice

Category	M2a markers	Host expression in response to IL-4		References
		Human	Mice	
C-type lectin receptors	MRC1 (CD206)	✓	✓	[20, 23, 24]
	MGL (CD301)	✓	✓	[6, 7, 26, 29, 30]
Enzymes	Arg1		✓	[34–36]
	TG-2	✓	✓	[23, 43]
Secreted proteins	FIZZ1 (RENTLA)		✓	[23, 50]
	Ym1 (CHI3L3)		✓	[23, 36]
Chemokine ligands	CCL17, CCL22	✓	✓	[4, 5, 63]
Cytokines	IL-10	✓	✓	[4, 7, 8, 29]
	TGF-β1	✓	✓	[4, 5, 8]
	IL-1RA	✓	✓	[5, 102–104]
Signal transduction proteins and transcription factors	STAT6, KLF4, SOCS1, and IRF4	✓	✓	[23, 126, 130, 134]

M2a markers; that are mentioned in this review; are divided into six major categories according to their nature. The host expression of human and mice is included

## M2a markers and allergic asthma

### C-type lectin receptors

#### Mannose receptor C type 1 (MRC1, CD206)

**Fig. 1:**
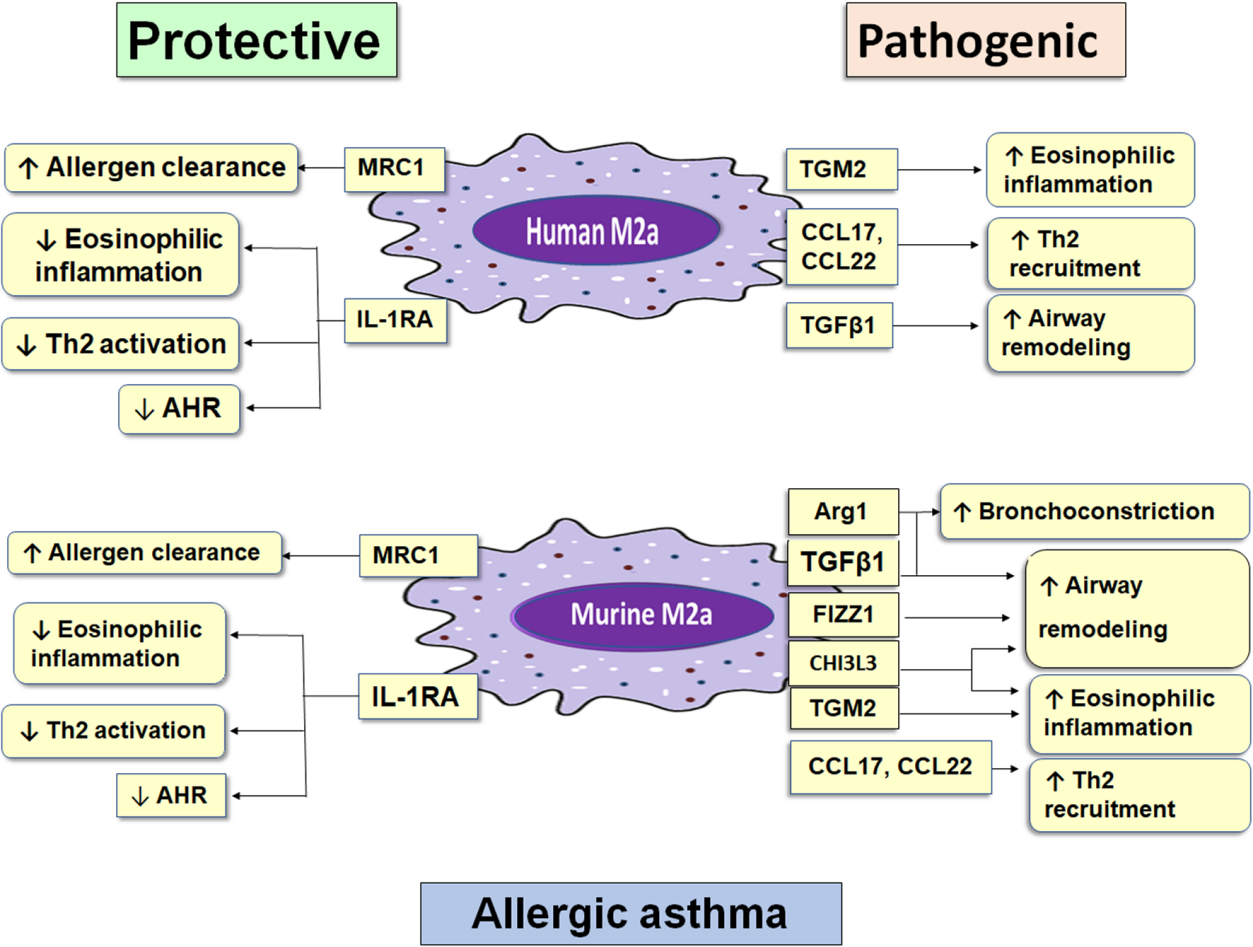
Protective and pathogenic proteins of human and murine M2a macrophages in allergic asthma. The protective proteins are the same in human and mice, representing in MRC1 that mediate allergen clearance, and IL-1RA that suppresses eosinophilic inflammation, Th2 activation and AHR, which induced by IL-1. The pathogenic proteins of human and mice are shared in three markers, TGM2 that induces eosinophilic inflammation, CCL17 and CCL22 that induces Th2 mediated allergic inflammation, and TGF β1 that induce airway remodeling. Murine M2a macrophages have another three unique pathogenic proteins, Arg1 that stimulate bronchoconstriction and airway remodeling, FIZZ1 that induces airway remodeling and finally CHI3L3 that also induces Airway remodeling and eosinophilic inflammation

Mannose receptor C type-1 (MRC1, CD206) are pattern recognition receptors and member of the C-type lectin receptor family expressed by macrophages and dendritic cells. MRC1 recognizes some terminal sugars of microorganisms such as *N*-acetyl-d-glucosamine, l-fucose and d-mannose, that are not expressed by eukaryotic cells. Therefore, these terminal sugars are considered pathogen-associated molecular patterns [20, 21]. This recognition process is the first step in the phagocytosis of bacteria, fungi, parasites, viruses, and allergens [21, 22]. MRC1 is a significant marker in alternative activation of macrophages in both human and mice [20, 23, 24]. In murine allergic asthma, MRC1 knockout mice display a significant reduction in the uptake and clearance of allergens by macrophages, together with exacerbated peri-bronchial inflammation and goblet cell hyperplasia. In addition, eosinophils numbers, levels of IL-4, IL-13 and allergen-specific IgE also significantly increase. Therefore, MRC1 has a protective role in allergic asthma which is mediated by allergen uptake and clearance (Fig. 1), and this function may be mediated through miR-511-3p; an intronic miRNA encoded by both mouse and human Mrc1/MRC1 genes [25].

#### Macrophage galactose type C-type lectin receptors (MGL/CD301)

Macrophage galactose type C-type lectin receptors (MGL/CD301) are pattern recognition receptors that recognize terminal galactose antigens. MGL are expressed on human (hMGL) and mice macrophages (mMGL), but mice have two types namely 1 and 2 (mMGL1 and mMGL2), with 60% homology between hMGL and mMGL1 [26–28]. IL-4 upregulates their expression, hence it is considered one of the M2a cell markers [6, 7, 26, 29, 30]. The role of MGL in allergic asthma is poorly understood. However, many potent allergens that induce asthma, have galactose terminals such as cockroach [31] and cat [32] allergens. Therefore, further studies are required to understand the role of MGL receptors in the immune response to these allergens.

### Enzymes

#### Arginase-1 (Arg1)

Arginase-1 (Arg1) is an enzyme that eliminates nitrogen by the hydrolysis of l-arginine into l-ornithine and urea, and is expressed in liver cells (human and mice) [33]. Murine macrophages’ Arg1 is considered one of the best described signature markers of AAM, unlike human macrophage that do not express Arg1 in response to Th2 cytokines [34–36]. l-arginine has two possible metabolic pathways. The first is catalyzed by inducible nitric oxide synthetase (iNOS) to NO and l-citrulline, which are characteristic of classical activation of macrophages. NO has a bronchodilator effect on airway smooth muscle cells [34]. The second pathway is catalyzed by Arg1 to l-ornithine as mentioned above. The balance between iNOS and Arg1 is required to maintain the normal muscle tone, and this explains a possible role of elevated Arg1 expression during asthma and how it stimulates bronchoconstriction [34, 37]. Thus, blocking of arginase pathway has been developed as a therapeutic target to direct l-arginine to iNOS pathway for the synthesis of the more bronchodilator NO [38]. Moreover, l-ornithine; the product of Arg1 pathway; is converted by ornithine decarboxylase to putrescine, which is converted by spermidine synthase and spermine synthase to spermidine and spermine, respectively [37]. In allergic asthma, the levels of l-ornithine derived polyamines are elevated. They increase the AHR to allergen, while AHR decreases with the treatment by inhibitors of polyamines’ synthesis [37]. Also, polyamines stimulate the contraction of smooth muscle cells through its effect on intracellular calcium [39]. In addition, l-ornithine is converted by ornithine aminotransferase to proline [40], a precursor of collagen. Therefore, elevated proline induces collagen depositions leading to airway remodeling [37, 41]. All these factors indicate the pathogenic roles of Arg1 in murine allergic asthma.

#### Transglutaminase 2 (TGM2)

Transglutaminase 2 (TGM2) induces structural modification of proteins by catalyzing the binding of low molecular weight primary amines. TGM2 is, also, involved in cell adhesion, migration and extra-cellular matrix regulation. TGM2 is synthesized in the cytoplasm of macrophages, then transported to the cell surface to bind heparan proteoglycans [42]. TGM2 is considered a new marker of IL-4-induced macrophages in both human and mice [23, 43]. In asthma, TGM2 potentiates the enzymatic activity of secreted phospholipase A2 (PLA2) group X (sPLA2-X), which in turn regulates the production of inflammatory cysteinyl leukotrienes (CysLT; eicosanoids) by mast cells and eosinophils [44, 45]. A member of cysteinyl leukotrienes; CysLT E4 induces the recruitment of eosinophils and basophils to the inflammatory sites, increases airway hyperresponsiveness and vascular permeability [46, 47]. In TGM2 knockout mice; the airway inflammation and hyperresponsiveness are attenuated, eosinophilic recruitment is reduced, and Th-2 differentiation and its main cytokines (IL-4 and IL-13) are suppressed together with the allergen-specific Ig-E. Also, the expression of IL-33 is decreased. In addition, the same findings are observed in allergic asthma of normal mice when treated with the TGM2 inhibitor (Cysteamine) [48]. These studies highlight the significance of TGM2 as a pathogenic factor in allergic asthma.

### Secreted proteins

#### Found in inflammatory zone 1 (FIZZ1)

Found in inflammatory zone-1 (FIZZ1) is a cysteine rich secreted protein known as resistin-like molecules (RELMs) and resistin-like alpha (RETNLA) discovered in 2000 [49]. FIZZ1 has no human homolog and is considered one of the signature markers of alternative activation of murine macrophages [23, 50]. FIZZ1 expression is upregulated during murine allergic asthma [49], and stimulates myofibroblasts hyperplasia in lung. Myofibroblasts are the major producers of Collagen Type I and α-smooth muscle actin (α-SMA). Myofibroblasts hyperplasia leads to collagen deposition in bronchial walls, therefore, inducing narrowing of airway passages and limitation of air movements. This phenomenon is called airway remodeling (Fig. 1) in asthma [51]. FIZZ1 knockout mice display a significant reduction in pulmonary fibrosis when treated with bleomycin (fibrosis-inducing agent). Conversely, FIZZ1 overexpression using a viral vector, exacerbates pulmonary fibrosis, thus confirming the profibrogenic role of FIZZ1 [52].

#### Chitinase 3-like 3 (CHI3L3, Ym1)

Chitinase 3-like 3 (CHI3L3); also called Ym1; is a secreted protein that lacks enzymatic activity of chitinase (hence it is name) that hydrolyzes the glycosidic bond in chitin; a polysaccharide component of fungal walls of and helminths. Despite lacking the enzymatic activity that can protect against pathogens, CHI3L3 can bind to chitin with high affinity and may recognize a pathogen-associated molecular pattern, although this role remains unclear [53]. CHI3L3 is expressed only in mouse with no human ortholog, and is considered one of the signature markers of murine AAM [23, 36]. In murine allergic asthma, CHI3L3 is highly expressed, correlated to levels of IL-4 and IL-13 [54], binds to carbohydrates e.g. heparin/heparan sulfate proteoglycan; which are major contributors of pulmonary fibrosis (by stimulating TGF-β signaling in fibroblasts) [55]. This suggests a possible role of CHI3L3 in airway remodeling of allergic lung [56]. Moreover, CHI3L3 recruits eosinophils to the inflammatory sites, and is considered as eosinophil chemotactic factor [57, 58]. Furthermore, this recruitment may be mediated through binding with heparan sulfate proteoglycan [59] suggesting the inflammatory and profibrotic roles of CHI3L3 in murine allergic asthma.

### Chemokines

#### CCL17 and CCL22

CCL17; also known as Thymus and activation-regulated chemokine (TARC) [60]; and CCL22; also known as macrophage-derived chemokine (MDC) [61]; are known ligands of CCR4, which is highly expressed by Th2 cells [5, 62]. Both CCL17 and CCL22 are considered human and murine M2a markers [4, 5, 63]. They chemoattract Th2 cells [64]. Their expression is upregulated in allergic asthma together with CCR4 expression to recruit more Th2 cells after allergen exposure [64, 65]. Also, CCL17 and CCL22 induce naïve T cell differentiation into Th2 cells [66, 67], indicating the pathogenic role of CCL17 and CCL22 in allergic asthma (Fig. 1). In addition, CCR4 blockade decreases AHR, eosinophilia, Th2 cytokines and their recruitment [64, 68] representing an effective target for asthma treatment. However, the blockade doesn’t completely abolish Th2 recruitment. CCR8 is thought to induces the recruitment [65]. However, in CCR8 deficient mice, Th2 cytokines and eosinophilia are not affected [69], suggesting the superiority of CCR4 in Th2 recruitment.

### Cytokines

#### IL-10

IL-10 is a potent immunosuppressive cytokine that is predominantly secreted by macrophages. It is, also, secreted by Th2, T regulatory cells (Treg), T cytotoxic, regulatory B lymphocytes, dendritic cells (DC), monocytes and mast cells. Its activity is mediated through IL-10 receptors, which belongs to type II cytokine receptors that also include IFN receptors. IL-10 suppresses macrophage MHCII, CD80 and CD86 expression. Thus, M1 features including antigen presentation, and secretion of proinflammatory cytokines (IL-12, IL-1β and TNF-α) are suppressed, which in turn inhibits Th1 activation. Also, IL-10 is the first known protein to inhibit IFN-γ, and is considered a negative feedback regulator of macrophages [70, 71]. In addition, IL-10 is one of the significant markers of M2a cells in human and mice [4, 7, 8, 29]. In allergic asthma, IL-10 level is elevated in serum [72], but decreases in bronchoalveolar lavage (BAL), which may reflect the binding of IL-10 to its receptors [73]. This suggestion is supported by increased IL-10 mRNA in BAL cells of asthmatic patients [74]. IL-10 is thought to suppress Th2 cytokines [75–77]. This suppression is mediated through induction of granzyme B that causes Th2 cell death [77] and also through inhibition of allergic antigen presenting functions and migration of DC to local lymph nodes [78]. IL-10, also, decreases eosinophilia [75–82] by suppressing their survival [79] or by reducing IFN-γ production; which ameliorates eosinophilic recruitment [75], and by suppressing Th2 cytokines. In addition, the high levels of IL-10 in serum of asthmatic patients are associated with lower risk of asthmatic exacerbations [83]. Interestingly, steroids exert some of their anti-inflammatory activity through stimulation of IL-10 secretion [73, 84]. Also, allergen-specific immunotherapy inhibits AHR and airway inflammation through IL-10 and Treg cells [85]. IL-10 suppresses total IgE [77, 80], although it doesn’t affect allergen-specific IgE [80, 81], despite suppressing IL-4 (the main inducer of IgE), which indicates that IL-4 might be less important in the late phase of the IgE production [80]. Surprisingly, IL-10 knockout mice have no or weak AHR in response to allergen [82, 86], which is supported by reconstitution of AHR upon increased expression of IL-10 [86]. This reaction might be a result of exaggerated inflammatory response to different exogenous stimuli in the absence of IL-10 [80]. Furthermore, IL-10 treatment induces AHR to allergen together with suppression of eosinophilia, which are paradoxical responses, due to the role of eosinophil in induction of AHR. However, this role is a controversial. Interestingly, this AHR might be a result of suppression of IFN-γ, which stimulates smooth muscle relaxation through β adrenergic receptors, or because IL-10 stimulates monocyte chemoattractant protein-1, which in turn induces histamine release from mast cells and basophils, leading to increased bronchial contractile activities [76]. In contrast to these finding, intratracheal administration of IL-10 ameliorates allergic AHR [81]. So, the precisive relationship between IL-10 and AHR together with IL-10 direct action on smooth muscle cells and indirect action through stimulation of other mediators, needs further studies. Another surprising finding about IL-10 role in allergic asthma, is the induction of airway fibrosis through IL-13/STAT6 pathway or increased TGF-β production, with mucus hypersecretion [87]. Finally, IL-10 has pleiotropic functions in critical features of asthma (ameliorate airway inflammation, induce AHR and remodeling), coupled with different regulators of its expression and actions, which raise the controversy, and make the interpretation account for additional challenges. Therefore, further studies of the roles of IL-10 in asthma are required, taking in consideration, the impact and cross talk of other regulators, for better understanding of IL-10 roles in allergic asthma.

#### TGF-β1

TGF-β1 is member of TGF-β family that was first identified as a tumor growth factor in vivo. This family includes three proteins; TGF-β1, TGF-β2 and TGF-β3. TGF-β1 is produced mainly by immune cells such as macrophages, Treg, eosinophils and many other cell types. TGF-β1 receptor is formed of two different proteins (TGF-βRI and TGF-βRII). Their binding stimulates phosphorylation of SMAD2 and SMAD3 transcription factors, which translocate to the nucleus and bind to the promotor of target genes [88, 89]. TGF-β1 inhibits M1 polarization [90], and is considered a marker of M2a cells in both human and mice [4, 5, 8]. Also, it controls the differentiation of Treg and Th17 cells [88, 89], stimulates IgA secretion by B cells, and promotes collagen synthesis and angiogenesis, which contribute to both tissue repair and fibrotic diseases [88]. TGF-β1 ensures normal branching and cellular differentiation of the lung during embryonic development [89].

In allergic asthma, TGF-β1 airway expression is increased [91–93] and correlates with the severity of asthma [93]. The actual impact of TGF-β1 on Th2 cells and eosinophils in allergic asthma is not well established. Some studies revealed that TGF-β1 neutralizing antibody diminished Th2 response together with eosinophilic infiltration [94], while others didn’t detect any correlation between TGF-β1 and Th2-mediated eosinophilic inflammation [91]. However, the main action of TGF-β1 is the induction of fibrosis and airway remodeling. TGF-β1 is considered one of the potent inducers of epithelial–mesenchymal transition (EMT), which promote myofibroblasts derived from bronchial epithelial cells, mediated through phosphorylated SMAD3 signaling pathway [95, 96]. Myofibroblasts express α-SMA, which mediate the contractile activity of fibroblastic cells, in addition to their production of collagen types I, III, IV and V [97]. Moreover, TGF-β1 increases contractility, migration and proliferation of airway smooth muscle cells [95, 98]. Therefore, it is not surprising that TGF-β1 correlates with the airway narrowing and limitation of air movements in asthmatic patients [91]. Thus, the potent contributions of TGF-β1 in airway remodeling and pulmonary fibrosis, make it an effective target to attenuate these effects by either specific microRNA [96] or specific inhibitors [99].

#### Interleukin-1 receptor antagonist (IL-1RA)

Interleukin-1 receptor antagonist (IL-1RA) is an anti-inflammatory cytokine and a member of the IL-1 cytokine family. It has 30% structural homology with IL-1β and 19% with IL-1α. IL-1RA binds to the same receptors (type I and type II IL-1R), but without activating any signal transductions (biologically inactive). Thus, it inhibits IL-1 actions. IL-1RA is the first detected natural cytokine inhibitor [100, 101]. There are two structural forms of IL-1RA; secretory and intracellular; both are produced by macrophages, while all other cells produce only one form except fibroblasts that produce both [101].

IL-1RA is one of M2a cell markers in both human and mice [5, 102–104]. In allergic asthma, IL-1 stimulates eosinophilic inflammation through induction of their recruitment by VCAM-1 expression [105, 106]. Also, it increases Ig-E dependent eosinophilic activation [107]. IL-1 activates Th2 cells with production of higher IL-4 (stimulates more Ig-E), IL-5 (induces eosinophilia) and IL-13, which also exacerbate AHR [108]; Fig. 1. This activation is mediated through IL-1 induction of OX40 (CD134) expression on T cells [109]. Moreover, IL-1 stimulates the secretion of platelet-derived growth factor which induces fibroblast proliferation and subsequent collagen synthesis, resulting in airway remodeling [110]. Indeed, all these actions of IL-1 are inhibited by IL-1RA, through its ability to bind to IL-1R and competitively inhibits the binding of IL-1. This inhibition is confirmed in IL-1RA knockout asthmatic mice, where Th2 cell activation and AHR are increased significantly compared with wild type [108]. In addition, the high levels of IL-1RA in asthmatic patients are associated with lower risk of asthmatic exacerbations [83]. Interestingly, glucocorticoids exert some of its action through inhibition of IL-1 secretion together with upregulation of IL-1RA expression [111, 112], which indicates the protective role of IL-1RA in asthma and its efficiency as a candidate for asthma treatment [113]. However, the use of recombinant IL-1RA as a therapy is limited with its short half-life and the fact that IL-1 is 100–1000 times more potent than IL-1RA, indicating the need for higher doses of IL-1RA to inhibit IL-1 actions, which may be accompanied with undesirable side effects [114, 115]. To overcome these limitations, the use of recombinant Adeno-virus expressing human IL-1RA is applied, through single intranasal administration in asthmatic mice, which proved its efficiency in ameliorating AHR and eosinophilic infiltration [116].

### Macrophage signal transduction proteins and transcription factors

We prefer to discuss the role of macrophage signal transduction proteins and transcription factors in allergic asthma through general potentiation of M2 cell characteristics. Since major transcription factors are, also, controlling Th2 cell polarization as STAT6 [117, 118], IRF4 [119, 120] and SOCS1 [121], we are concerned in this section with their role in macrophage polarization only, not generally in allergic asthma. Eventually, the transcription factors will contribute to the previously mentioned roles of other markers in asthma.

#### STAT6

STAT6 is a macrophage transcription factor and member of the STAT family that includes seven members (STAT1 to 4, 5a, 5b and STAT6). This family; together with non-receptor tyrosine kinase called JAK family; comprise the signal transduction pathway for type I and type II cytokine receptors [122, 123]. Binding of IL-4 or IL-13 to their receptors on macrophages activates JAK, which in turn phosphorylates the tyrosine residue on of IL-4Rα or IL-13Rα. This phosphorylation leads to recruitment of monomeric STAT6, which binds through its Src homology 2 (SH2) to phosphorylated tyrosine. Therefore, STAT6 and JAK are close to each other, the latter phosphorylates STAT6. The phosphorylated SH2 domain of monomeric STAT6 can bind to adjacent SH2 domain of another STAT6, forming a dimeric STAT6 that migrates to the nucleus, binds to specific promoters, inducing M2 genes transcription in human and mice [23, 122, 124]. Moreover, STAT6 activates Krüppel-like factor 4 (KLF4) that performs a crucial role in M2 cell polarization as discussed below.

#### Krüppel-like factor 4 (KLF4)

Krüppel-like factor 4 (KLF4); also called gut-enriched Krüppel-like factor or GKLF; is a DNA-binding transcription factor that contains conserved zinc fingers. It regulates various cellular processes such as differentiation, growth and proliferation. KLF4 was extensively studied particularly after 2006, since it was one of four factors required for pluripotent stem cells induction [125]. KLF4 is upregulated in macrophages in response to IL-4 in both human and mice [23, 126]. KLF4 and STAT6 activate each other, then activated KLF4 induces RNase and deubiquitinase activities of monocyte chemoattractant protein induced protein (MCPIP), which stimulates reactive oxygen species production. The latter causes endoplasmic reticulum (ER) stress and autophagy required for M2 cell polarization and upregulation of its markers. In addition, activated KLF4 stimulates the multifaceted factor peroxisome proliferator-activated receptor γ (PPARγ) that regulates fatty acids metabolism inducing aerobic respiration, which is necessary for M2 cell differentiation [127]. Moreover, MCPIP inhibits M1 polarization through inhibition of NF-κB pathway [127, 128].

#### Suppressor of Cytokine Signaling 1 (SOCS1)

Suppressor of Cytokine Signaling-1 (SOCS1) is a member of SOCS proteins that are responsible for negative feedback regulation of JAK–STAT signaling pathways, which transduces the signals from type I and II cytokines receptors. SOCS1 binds to phosphorylated STATs and JAKs, then, the tightly associated E3 ligases ubiquitinate the JAKs and STATs targeting them for degradation by proteasome [129]. SOCS1 is expressed in both human and mice macrophages in response to IL-4 stimulation [23, 130]. SOCS1 suppresses IFN-γ induced JAK2/STAT1 pathway and TLR/NF-κB signaling, which in turn lead to inhibition of M1 cell activation [129, 131]. On the other hand, micro RNA-155 (miR-155) binds to and degrades SOCS1 in M1 polarized macrophages [132, 133]. Moreover, SOCS1 is a crucial factor for M2 cell polarization, by enhancing PI3K activity, which is responsible for M2 cell characteristics including suppressed response to IFN-γ/LPS and high Arg1:iNOS activity ratio [129–131].

#### Interferon regulatory factor 4 (IRF4)

Interferon regulatory factor 4 (IRF4) is a transcription factor that belongs to the IRF family, which includes nine members. This family is involved in macrophage polarization [134]. IRF4 is upregulated in macrophages by IL-4 in both human and mice [23, 134]. Stimulated IRF4 reciprocally activates the histone demethylase Jumonji domain containing-3 (Jmjd3). The latter removes the methylation of histone H3 Lys4 (H3K4) and histone H3 Lys 27 (H3K27) that mediate silencing of M2 marker genes. Thus, this methylation removal induces expression of M2 marker genes such as Arg1, MRC1, Ym1 and FIZZ1. It, also, inhibits the polarization of M1 cells [134].

Some studies suggested that targeting macrophage transcription factors might ameliorate murine asthmatic inflammation through suppression of M2 cell polarization, which was performed by using specific inhibitor (protostemonine) that inhibits STAT6, KLF4 and IRF4. This reflected the critical role of M2 cells in asthma [135]. However, their evidences are not enough to prove the efficiency of targeting M2 cells in treatment of—nor their role in asthma, because STAT6 is also a crucial transcription factor for Th2 cell polarization [117, 118]. In addition, IRF4 controls Th2 polarization [119, 120]. Thus, the suppressive effect of protostemonine in asthma was mediated through inhibition of Th2 differentiation mainly. So, further studies are required for elucidating the specific roles of M2 transcription factors in the pathogenesis and treatment of asthma.

The above data show that human and murine M2a macrophages can mediate their protective function in allergic asthma through MRC1 and IL-1RA proteins. At the same time, Human M2a cells could have a pathogenic role through TGM2, CCL17, CCL22 and TGFβ1. However, murine M2a cells pathogenic functions are mediated through unique molecules as Arg1, FIZZ1 and CHI3L3 plus the previously mentioned human mediators (TGM2, CCL17, CCL22 and TGFβ1). Therefore, we think that AAM is a double-edged sword in allergic asthma.

## Summary, conclusion and recommendations

The expressed proteins of IL-4 activated alveolar macrophages perform diverse functions in allergic asthma, ranging between protective and pathogenic roles (Fig. 1). This balanced function not necessarily represent the non-significance of AAM in allergic asthma, but both protective and pathogenic molecules are important during developments of asthma. Therefore, targeting the general polarization of M2 cells, whether with more activation or inhibition, is not an efficient option for asthma treatment. However, selective induction of the expression of one or more of the protective molecules, or selective suppression of one or more of the pathogenic molecules, using viral vectors or other methods, represents an effective mechanism for asthma control and treatment. Thus, we recommend further studies that focus on increasing the selective expression of protective M2a proteins such as MRC1 and IL-1RA. Also, we recommend selective suppression of pathogenic M2a proteins e.g. TGM2, CCL17/CCL22, TGF-β1, and murine Arg1, FIZZ1 and CHI3L3, for the future developments of effective therapies for allergic asthma.

## Data Availability

All data pertinent to this manuscript are included herein

## Abbreviations

α-SMAα: Smooth muscle actin
AAM: Alternatively activated macrophages
AHR: Airway hyperresponsiveness
Arg1: Arginase-1
BAL: Bronchoalveolar lavage
CHI3L3/Ym1: Chitinase 3-like 3
FIZZ1: Found in inflammatory zone 1
IRF4: Interferon regulatory factor 4
KLF4: Krüppel-like factor 4
MGL/CD301: Macrophage galactose type C-type lectin receptors
MRC1/CD206: Mannose receptor C type 1
SOCS1: Suppressor of Cytokine Signaling 1
Treg: T regulatory cells
TGM2: Transglutaminase 2
